# Droplet Transmission of Streptococcus sanguinis Causing Pyogenic Liver Abscess in a Male Patient With a Transplanted Liver

**DOI:** 10.7759/cureus.71228

**Published:** 2024-10-10

**Authors:** Douaa Abou Hamdan, Farah Assi, Nada Chamseddine

**Affiliations:** 1 Infectious Diseases and Internal Medicine, Lebanese University, Beirut, LBN; 2 Infectious Diseases and Internal Medicine, Al Zahraa Hospital University Medical Center, Beirut, LBN

**Keywords:** bacteremia, biliary drain, droplet transmission, liver transplant, pyogenic liver abscess, streptococcus sanguinis

## Abstract

*Streptococcus sanguinis *is abundantly present in the oral cavity and colonizes the enamel as a natural part of the microbiome. Through the formation of a healthy biofilm, this bacterium provides a protective role. However, under certain conditions, it has the ability to become pathogenic. A pyogenic liver abscess (PLA) occurs through the contiguous spread of infectious pathogens from neighboring structures or following a trauma. The isolation of *S.* sanguinis as the pathogen in liver abscesses has only been reported twice. Here, we report the third case of PLA caused by *S. sanguinis* occurring in a young adult male patient, with a transplanted liver, biliary stenosis, and external drain. The bacterium was isolated from blood cultures. The most likely route of the acquisition was droplet transmission of a family member during drain irrigation. The patient was treated successfully with antibiotics, and he fully recovered.

## Introduction

*Viridans *group streptococci are natural commensals of the human microbiome, most predominantly found in the oropharynx, as well as in the gastrointestinal and genitourinary tracts [1]. *Viridans* group streptococci constitute a genetically heterogeneous group of non-beta-hemolytic streptococci [2]. *Streptococcus sanguinis* is a catalase-negative gram-positive coccus classified within the *S. mitis* group, based on 16S RNA sequence analysis, belonging to the *viridans* group [2,3]. Upon culturing on blood agar, this bacterium produces a greenish discoloration, thus demonstrating what is referred to as alpha hemolysis [3]. Various studies have demonstrated the importance and the protective role that *Streptococcu*s *sanguinis* confers through its ubiquitous and abundant presence in the oral cavity and the formation of a healthy biofilm on the surfaces of the teeth [4,5]. However, this commensal organism involved with healthy oral flora may shift to become pathogenic under certain conditions, such as in individuals who have undergone dental procedures, and those with poor oral hygiene, cancer, hematological malignancies, immunosuppression with chemotherapy, mucosal barrier disruption, and neutropenia [1,2,5].

A pyogenic liver abscess (PLA) is a purulent infection of the liver parenchyma most commonly caused by a bacterial etiology [6]. The recorded annual incidence of liver abscesses has varied across countries, with rates of 1.1-2.3 cases per 100,000 per year reported in Canada and Denmark, and up to 17.5 per 100,000 in Taiwan and 5-20 per 100,000 in the USA [7]. The microbiology of liver abscesses is variable across studies, reflecting etiological and demographic differences. Streptococcus species are being increasingly recognized as causative microorganisms, and in one population-based study, they were found to be the most commonly reported bacterial group (29.5%) [8]. Here we report a case of liver abscess occurring post-liver transplant and biliary instrumentation with isolation of *S. sanguinis* in blood cultures.

## Case presentation

We report the case of a 27-year-old male who is known to have rapidly progressive primary sclerosing cholangitis (PSC). Diagnosed in 2021, after suffering from recurring episodes of obstructive cholangitis, the patient eventually underwent partial living-donor liver transplantation with hepaticojejunal anastomosis in another country. His postoperative course was complicated by ischemic biliary disease and biliary stricture, which were managed by external drainage. Afterward, the patient repeatedly suffered from recurrent biliary obstruction that required treatment by irrigation through the external drain. The irrigation was performed three times per day, as advised by his surgeon. He was maintained on immunosuppressive medications tacrolimus (Prograf), everolimus (Certican), and steroids.

The patient presented to the emergency department of our hospital complaining of a high-grade fever, reaching up to 39°C and chills, accompanied by nausea, vomiting, and dark urine for the last three days. He has already been taking ciprofloxacin tablets (500 mg twice daily) for the last three days since the fever started without consulting a physician. Upon presentation, the patient was found to be febrile, with a temperature of 39.9°C, and hypotensive, with a blood pressure (BP) of 80/45 mmHg. His pulse rate was 125 beats/min, and his oxygen saturation was 97%. The physical exam showed a pale and lethargic patient, with an intact neurological examination. The abdomen was soft with a mild right upper quadrant tenderness. Auscultation of the heart and lungs revealed no abnormalities. He was admitted to a medicine ward for possible biliary infection. The patient’s blood pressure normalized after immediate fluid boluses and empirical antibiotics and did not require the use of vasopressors. He was started on piperacillin/tazobactam intravenously (IV) at a dose of 4.5 g every eight hours for likely intra-abdominal infection, after two sets of blood cultures, as well as fluid culture from the percutaneous biliary drain, were obtained.

Initial hematologic and biochemical laboratory data demonstrated an elevated gamma-glutamyl transpeptidase (GGT) and liver enzyme tests, mild leukocytosis, a high C-reactive protein (CRP), and an acute kidney injury, as listed in Table 1.

**Table 1:** Hematological and biochemical laboratory data at admission WBC: white blood cells; Hb: hemoglobin; Plt: platelets; Cr: creatinine; C-RP: C-reactive protein; AST: aspartate transaminase; ALT: alanine transaminase; GGT: gamma-glutamyl transpeptidase; Alk Ph: alkaline phosphatase; T-Bil: total bilirubin; D-Bil: direct bilirubin

Parameter		Value	Reference range
WBC		11.25x10³/µL	4.0-11.0
Neutrophils		80%	40-65
Hb		16 g/dL	13-18 (male)
Plt		142000/µL	150-400
Cr		1.44 mg/dL	0.67-1.17
C-RP		45 mg/L	0-5
AST		46 U/L	5-41
ALT		63 U/L	5-41
GGT		296 U/L	5-61
Alk Ph		142 U/L	35-129
T-Bil		0.85 mg/dL	0.2-1.0
D-Bil		0.29 mg/dL	0.0-0.2
Urine analysis: WBC’s		6-8/hpf	0-5
Urine Analysis: RBC’s		2-3/hpf	0-5

**Figure 1:**
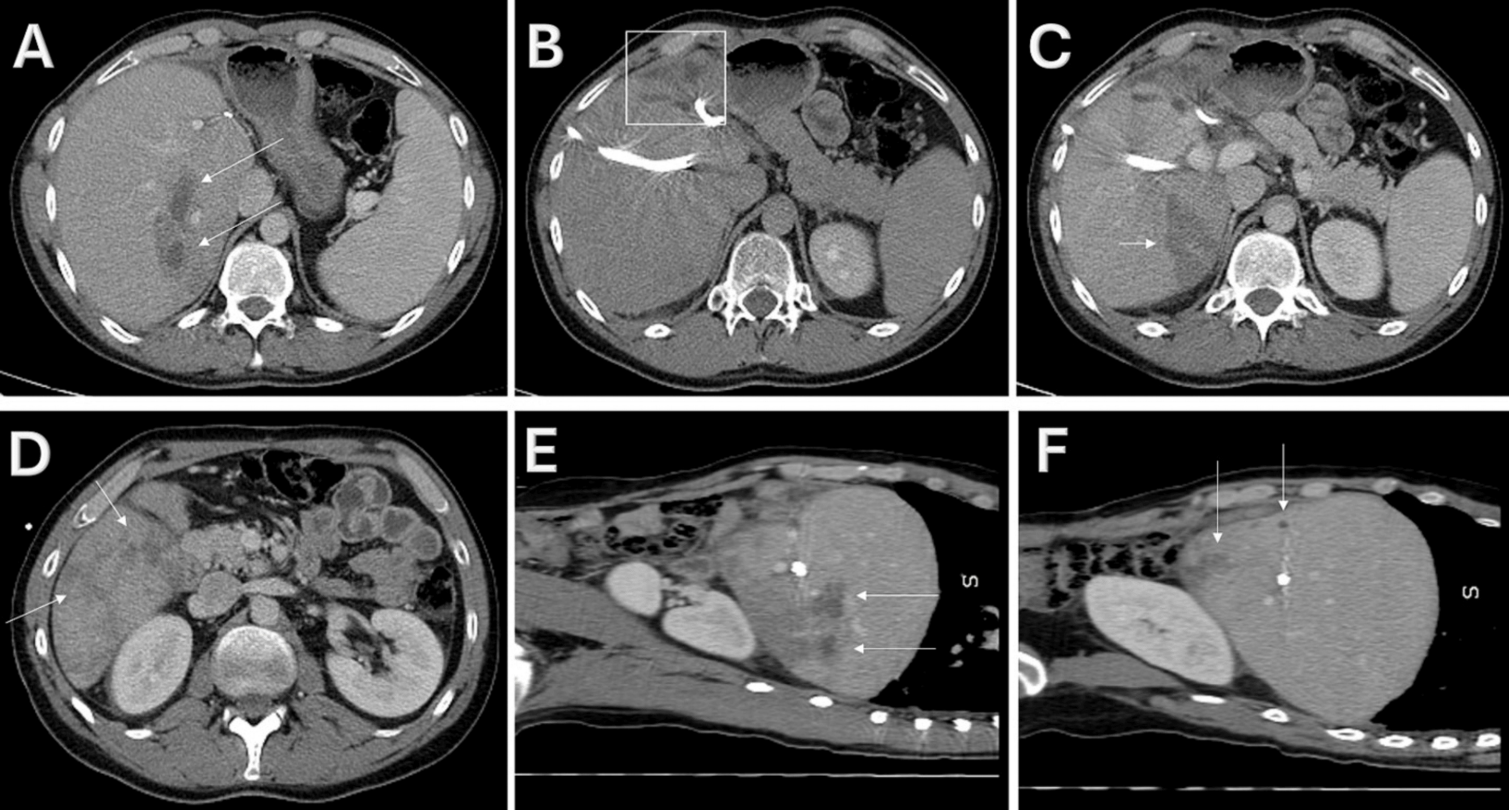
CECT scan of the abdomen (portal venous phase) showing multiple small hepatic abscesses A shows two abscesses (arrows) in segments VII and at the junction of VII and VIII; B shows subcapsular small abscesses (squares) in segments IV-B and VIII, with extension to the subhepatic area; C and D show ill-defined hypodense areas (arrows) in segments V and VI; E and F show sagittal sections revealing multiple small liver abscesses (arrows). CECT: contrast-enhanced computed tomography

An ultrasound of the abdomen and the pelvis was performed revealing a congested and heterogeneous liver parenchyma. Moreover, it detected a hypoechoic formation (2.2 cm) with a necrotic center at the right lobe of the liver, suggestive of an abscess. After the patient’s creatinine level normalized following adequate hydration, a contrast-enhanced computed tomography (CECT) scan of the abdomen was performed. It demonstrated multiple small hypodense lesions in several liver segments (IV-B, V, VI, VII, and VIII) (Figure 1). These findings were suggestive of small liver abscesses. The sizes of these lesions were, unfortunately, not reported.

**Figure 2:**
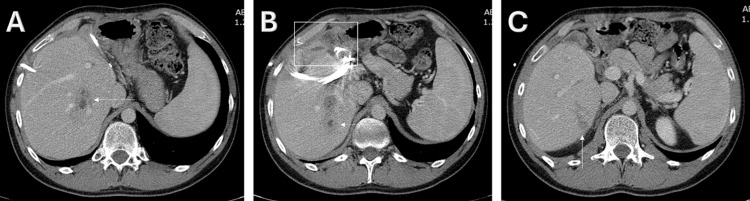
Follow-up CECT scan of the abdomen, after 12 days of antibiotics A: slight decrease in the size of the abscess at the junction of segments VII and VIII (arrow); B: slight decrease in the size of the abscess at segment VII (arrow), as well as in the subcapsular abscesses at segments IV-B and VIII (square); C: decrease in the extension of the ill-defined hypodense lesions previously seen in segments V and VI (arrow). CECT: contrast-enhanced computed tomography

Twenty-four hours after the initiation of antibiotics, the patient’s temperature resided, and the patient was showing an overall favorable clinical response. On the fourth day of admission, all four drawn bottles of blood cultures confirmed the growth of gram-positive cocci, identified later by the VITEK 2 system to be *S. sanguinis*. The bacterium was sensitive to erythromycin, ceftriaxone, cefepime, clindamycin, tetracycline, tigecycline, and vancomycin, according to the susceptibility testing. Piperacillin/tazobactam was then stopped after a total of four days, and IV ceftriaxone was started at a dose of 1 g every 12 hours. A transthoracic echocardiography, performed for the purpose of evaluating the need for further investigations to rule out infective endocarditis, revealed no cardiac abnormality and no suspicion of vegetation. After 12 days of treatment with IV antibiotics, a follow-up CECT scan of the abdomen revealed only a slight decrease in the sizes and the extension of the abscesses (Figure 2).

Follow-up labs at this time displayed resolution of the leukocytosis, a decrease in his CRP level (8 mg/L), and improvement of his transaminases (ALT 49 U/L and AST 29 U/L). The GGT only slightly decreased from the admission value (272 U/L). Since the abscesses were not accessible for percutaneous drainage, due to their small size and multitude, as discussed with the radiologist, the patient was discharged on oral amoxicillin for an additional four weeks and was planned for cholangiography.

## Discussion

PLA carries a high morbidity burden with a mortality rate estimated to range between 6% and 31% [9]. Hippocrates was the first to report a case of PLA in 400 BCE and the mortality rate was close to 100% in the pre-antibiotic era [8,10]. The formation of liver abscesses is a consequence of the inability of the inflammatory response to successfully eliminate the infectious agent [11]. Several pathological mechanisms have been proposed, which include biliary spread, hematogenous spread through the portal vein or hepatic artery, extension of infection from an adjacent area, injury from a penetrating trauma or a surgical wound, and occasionally, a migrating ingested foreign body [12]. Various predisposing risk factors associated with the development of PLA in non-liver transplant patients and liver transplant patients have been described, and they include diabetes mellitus, proton-pump inhibitor (PPI) use, prior liver surgery, hepatic malignancy, cholecystitis, and cholangitis in non-liver transplant patients [13]. In patients who have undergone a liver transplant, the risk factors are biliary stricture, hepatic artery thrombosis, diabetes mellitus, portal vein thrombosis, serum creatinine >1.5 mg/dL, Roux-en-Y hepaticojejunostomy procedure, and re-transplantation [14]. The different pathogens that have been implicated in the formation of liver abscesses can be categorized into three groups: parasitic (*Entamoeba histolytica*), fungal (candida), and bacterial [15,16]. Most PLA are polymicrobial, with the most commonly isolated pathogens being enteric gram-negative bacilli (*Klebsiella pneumoniae; Escherichia coli*), anaerobes, *Streptococcus milleri*, and *Staphylococcus aureus* [15]. In a case series study, streptococcus species were found to be the most commonly isolated group of organisms (29.5%) [10]. In patients who have undergone a liver transplant, one retrospective case-control study identified Enterobacteriaceae, Enterococcus species, and anaerobic bacteria as the most commonly isolated pathogens [17]. The most frequently reported manifestations are fever, chills, and right upper quadrant abdominal pain [7].

The patient in our case report had undergone a liver transplant and was maintained on immunosuppressive drugs (tacrolimus, everolimus, and steroids). His course was complicated by the occurrence of biliary stricture requiring the insertion of an external drainage, and frequent episodes of biliary obstruction. He was not a known diabetic; however, he did suffer from steroids-induced hyperglycemia for a short duration of time, which resolved without treatment upon tapering and stopping of steroids. Our patient has thus fulfilled many of the aforementioned risk factors for the development of liver abscesses. He presented with fever, chills, vomiting, and right upper quadrant tenderness, consistent with the signs and symptoms described in the literature. Imaging showed multiple small lesions suggestive of abscesses, which were inaccessible for drainage or aspiration due to their small size. Moreover, the four bottles of blood cultures yielded bacterial growth of* S. sanguinis*. Upon further questioning, the patient denied having had any dental procedures or issues since pre-transplantation, and throughout the period until presentation to the hospital. He reported that the frequent (three times per day) irrigation, advised by his surgeon, was being performed by his sister, without using a face mask or any other personal protective equipment, and she did so while continuously conversing with the patient. Thus, our theoretical assumption was that this act carries a high risk of oral droplet transmission of *S. sanguinis* from the sister’s oral cavity to the patient’s drain port. A similar mode of transmission of S. sanguinis and other alpha-hemolytic streptococci strains has been documented to occur following lumbar puncture in the stetting of performing the procedure without appropriate infection control precautions, including wearing face masks [18].

Existing naturally among the oral cavity flora, this bacterium has only rarely been associated with liver abscesses. To our knowledge, only two cases were previously reported that describe this association. The first case was reported by Shih et al., and dates back to 1985, when a male patient, aged 45 years, complained of epigastric pain, and fullness, followed by fever [19]. The physical exam revealed an enlarged and tender liver. The diagnosis of liver abscess was established using liver-spleen scintigraphy, which revealed a 10 cm mass in the posterior aspect of the right liver lobe. CT-guided percutaneous drainage of the abscess yielded the growth of* S. sanguinis*, treated by cefacitin, tobramycin, and metronidazole. No explanation regarding the origin of the *S. sanguinis* was provided by the authors. The patient recovered and was discharged with a good prognosis. The second case, reported by George et al. in 1996, is that of a 39-year-old male, who developed a fever with disturbed liver function tests [20]. CT imaging revealed the presence of an 8 cm liver mass in the anterior part of the right hepatic lobe with multiple hypoechoic areas. Ultrasonographic-guided aspiration of the lesion drained brownish purulent fluid that later yielded the pure growth of* S. sanguinis*. In-hospital treatment with penicillin G and metronidazole (1 week), followed by home treatment with IV ampicillin and oral metronidazole for three additional weeks led to complete resolution of the abscess. The occurrence of the liver abscess was linked to a gastric mucosal biopsy performed during an upper gastroesophageal endoscopy two months prior to presentation.

Due to a technical problem in our microbiology lab, the sensitivity analysis of *S. sanguinis* lacked penicillin susceptibility. However, the isolated germ was sensitive to the remaining associated antibiotics. Piperacillin/tazobactam was then stopped, and the patient was continued on ceftriaxone. He showed clinical improvement after 24 hours following antibiotic therapy. His course in the hospital was overall uncomplicated.

## Conclusions

Liver abscess carries a high mortality rate, with multiple risk factors implicated in its pathogenesis. We present a case of liver abscess associated with *S. sanguinis* bacteremia in the setting of liver transplant, immunosuppression, and biliary stricture with an external drain. *S. sanguinis* is an infrequently isolated germ in this condition, and transmission can occur while performing a medical act on a non-intact skin site, without the use of proper personal protective equipment. Treatment with antibiotics exhibits an overall good prognosis.
